# Structural and functional characterization of human kallistatin

**DOI:** 10.1016/j.bbrep.2026.102651

**Published:** 2026-06-02

**Authors:** Stephanie T.D. Pham, Kristian W. Nielsen, Jonas H. Graversen, Nanna Kristensen, Lasse B. Steffensen, Andrea R. Lundgaard, José L. Martín-Ventura, Peter Højrup, Yaseelan Palarasah

**Affiliations:** a Inflammation Research Unit, Department of Molecular Medicine, University of Southern Denmark, Odense, Denmark; b Department of Biochemistry and Molecular Biology, University of Southern Denmark, Odense, Denmark; c Cardiovascular and Renal Research Unit, Department of Molecular Medicine, University of Southern Denmark, Odense, Denmark; d Vascular Research Laboratory, IIS-Fundación Jiménez Díaz, Madrid, Spain; e CIBER de Enfermedades Cardiovasculares (CIBERCV), Madrid, Spain

**Keywords:** Kallistatin, Glycosylation, Polymerization, ELISA, Mass spectrometry, Abdominal aortic aneurysm

## Abstract

Kallistatin is a serine protease inhibitor (serpin) that specifically inhibits tissue kallikrein, a key enzyme involved in kinin generation and vascular homeostasis. While serpins are known to polymerize under certain conditions, the structural properties of human kallistatin—particularly its glycosylation profile, tissue distribution, and polymerization potential—remain poorly defined.

In this study, we generated a panel of kallistatin-specific monoclonal antibodies and developed a sensitive sandwich ELISA that enables reliable quantification of kallistatin in both plasma and tissue-conditioned media. The generated antibodies facilitated both quantitative and qualitative analyses, allowing detailed characterization of kallistatin's structural features, glycosylation profile, and localization in vascular tissue from patients with abdominal aortic aneurysm. Using mass-spectrometry, we characterized the glycosylation sites of kallistatin, providing experimental confirmation of the putative glycosylation at Asn 33.

Biochemical analyses revealed that kallistatin can polymerize and is susceptible to structural rearrangements typical of serpins. Deglycosylation markedly increased polymer formation demonstrating that glycosylation plays a critical stabilizing role in preventing polymerization. Functional assays using a fluorogenic tissue kallikrein substrate showed that polymerized kallistatin loses inhibitory activity, whereas deglycosylated kallistatin retains normal function. This indicates that glycosylation primarily supports structural stability rather than directly modulating inhibitory capacity.

These findings provide new insights into kallistatin's structural features, including its glycosylation, stability, and polymerization behavior, and establish essential tools for further exploring its physiological and pathological roles.

## Highlights

• Kallistatin specific mAbs provide insights into kallistatin's structural features.
• Experimental confirmation of glycosylation at Asn 33 in kallistatin.
• Deglycosylation increases kallistatin's tendency to polymerize.
• Kallistatin polymerization abolishes its inhibitory function on tissue kallikrein.
• Glycosylation stabilizes kallistatin's structure rather than affecting function.

## Graphical abstract

**Figure:**
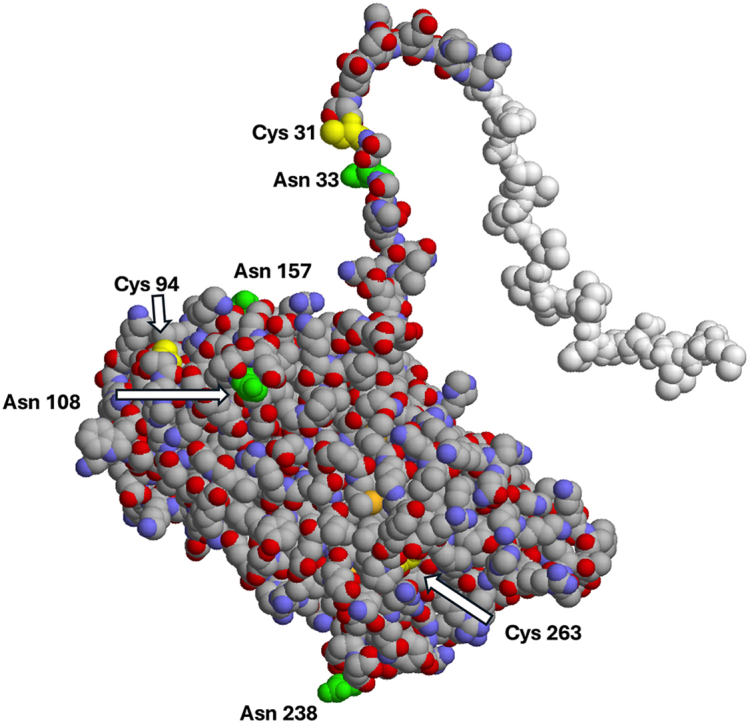

## Abbreviations

AAA: Abdominal aortic aneurysm
CNBr: Cyanogen-bromide
ELISA: Enzyme-Linked Immunosorbent Assay
FPLC: Fast protein liquid chromatography
HSA: Human serum albumin
HRP: Horseradish peroxidase
Ig: Immunoglobulin
mAb: Monoclonal antibody
PNGase F: Peptide-N-Glycosidase F
PNGase A: Peptide-N-Glycosidase A
Serpin: Serine protease inhibitor
SDS-PAGE: Sodium Dodecyl Sulfate-Polyacrylamide Gel Electrophoresis
SA-PE: Streptavidin-phycoerythrin
TFA: Trifluoroacetic acid
VSMCs: Vascular smooth muscle cells

## 1 Introduction

Kallistatin is a 58-60 kDa glycoprotein belonging to the serine protease inhibitor (serpin) superfamily [1]. It is primarily synthesized in the liver and secreted into the circulation [1,2], but it has also been detected in various tissues including the heart, vasculature, and kidneys [3,4]. Kallistatin functions as a specific inhibitor of tissue kallikrein, a protease involved in generating kinins from low-molecular-weight kininogen, which modulates processes such as blood pressure regulation, inflammation, and vascular tone [2,3,5]. Upon binding to tissue kallikrein, kallistatin forms a sodium dodecyl sulfate-polyacrylamide gel electrophoresis (SDS)-stable enzyme-inhibitor complex that irreversibly blocks kallikrein's proteolytic activity [6,7].

Structurally, kallistatin contains two key functional domains: a reactive center loop responsible for protease inhibition and a heparin-binding site [3]. Heparin binding interferes with the ability of kallistatin to inhibit tissue kallikrein by preventing complex formation [7]. The mature protein consists of 401 residues [4], and contains four potential N-linked glycosylation sites: Asn 33 (predicted), Asn 108, Asn 157, and Asn 238 [[8], [9], [10]]. Glycosylation plays a critical role in protein folding, stability, solubility, and prevention of protein aggregation [[11], [12], [13]].

Serpins in general have a tendency to polymerize, particularly under stressed conditions such as elevated temperature or structural destabilization [14,15]. For the serpin α_1_-antitrypsin, glycosylation has been shown to enhance the conformational stability and reduce the tendency to form pathogenic polymers [16]. This raises the possibility that altered glycosylations in kallistatin could influence its polymerization behavior and potentially contribute to disease mechanisms [[17], [18], [19], [20]]. However, the molecular characteristics of kallistatin in human tissues and plasma remain incompletely understood, particularly regarding its glycosylation status, tissue distribution, and polymerization potential.

Kallistatin has been implicated in a wide range of pathological conditions reflecting its pleiotropic roles in modulating inflammation, oxidative stress, angiogenesis, and apoptosis. Reduced kallistatin levels have been reported in patients with hypertension, sepsis, diabetic retinopathy, inflammatory bowel disease, liver disease, and various cancers, including prostate and colon cancer [3]. In cardiovascular disease, kallistatin has shown anti-atherogenic effects by reducing vascular inflammation, improving endothelial function, and modulating lipid metabolism [21]. In metabolic disorders, kallistatin has been linked to improved lipid handling and attenuation of cardiac hypertrophy via the SIRT1/PPARα pathway [22]. Moreover, kallistatin has been shown to influence cognitive decline in Alzheimer's disease models by promoting amyloid-β accumulation and tau phosphorylation, suggesting a mechanistic link between metabolic syndrome and neurodegeneration [23]. In autoimmune disease, kallistatin overexpression has been associated with exacerbation of experimental autoimmune uveitis through enhanced Th17 differentiation [24]. In the context of vascular disease, kallistatin has been proposed to exert protective effects in abdominal aortic aneurysm (AAA) by modulating inflammation, oxidative stress, and extracellular matrix remodeling [25].

In this study, we aimed to comprehensively characterize human kallistatin with respect to its plasma levels, presence in tissue-conditioned media, glycosylation status, and polymerization behavior. To support these investigations, we developed a panel of monoclonal antibodies (mAbs) against kallistatin, which were employed as key analytical tools throughout the study.

We established a sensitive and specific ELISA for the quantification of kallistatin in plasma. The presence of kallistatin in the local vascular environment was evaluated using tissue-conditioned media from AAA patients. Furthermore, native kallistatin purified from plasma, using the developed mAbs, and recombinant expressed kallistatin were characterized in detail in relation to glycosylation sites, glycan compositions, polymerization, and functional impact of its inhibitory effect on tissue kallikrein activity.

## 2 Materials and methods

### 2.1 Generation of human kallistatin specific monoclonal antibodies

NMRI mice were immunized twice subcutaneously with an interval of at least 14 days with 20 μg of recombinant kallistatin coupled to diphtheria toxoid via the terminal cysteine and adsorbed to Al(OH)_3_ and mixed in a 1:1 ratio with Freund's incomplete adjuvant. Fourteen days after the last injection and 3 days prior to the sacrifice, the mice received an intravenous boost with 20 μg of the coupled recombinant kallistatin. Fusion of the isolated spleen cells with myeloma cells to generate hybridoma cells, as well as the subsequent selection procedure, was essentially conducted as described by Köhler and Milstein [26], except that the SP2/0-AG14 myeloma cell line was used as fusion partner. Positive hybridoma clones were selected by screening against both in-house recombinant expressed kallistatin and commercial recombinant expressed kallistatin (R&D Systems, Minneapolis, MN, United States). Positive hybridomas were identified and cloned by the limited dilution method until single clones were obtained. Single clones were then grown in culture flasks in RPMI-1640 (Lonza, BioWhittaker®, Basel, Switzerland) supplemented with sodium pyruvate (Gibco™, Waltham, MA, United States) and gentamicin sulfate (Biowest®, Nuaillé, France) and contained 10% fetal bovine serum (Biowest®). The mAbs were purified from culture supernatants by Protein G affinity chromatography. The development of the mAbs were conducted under approval from the Danish Animal Experiments Inspectorate (Dyreforsøgstilsynet), which operates under the Ministry of Food, Agriculture and Fisheries of Denmark. Immunizations were performed in accordance with ethical approval (approval number 2015-15-0201-00680).

### 2.2 Kallistatin specific sandwich ELISA

A panel of kallistatin specific mAbs was produced, and after testing different capture–detection combinations, a matched pair of mAbs was selected to establish the kallistatin quantification assay. The kallistatin assay was constructed as a non-competitive sandwich ELISA using the mAb 22-21-7 as capture antibody. Biotinylated 22-21-9 was used as detection antibody. MaxiSorp plates (Thermo Fisher Scientific, Waltham, MA, United States) were coated and incubated overnight at 4 °C with 100 μL/well of 2 μg/mL mAb 22-21-7 diluted in coating buffer (5 mM Na2CO_3_, 35 mM NaHCO_3_, pH 9.6). The wells were washed three times in PBS, 0.05% Tween-20, pH 7.4 (PBS-TW) and blocked with PBS-TW/0.5% skimmed milk (Honeywell Fluka™, Charlotte, NC, United States) for 30 min at RT with agitation to block remaining reactive sites. A pool of EDTA plasma served as a standard and calibrator, and to estimate the kallistatin concentration in this standard, recombinant kallistatin (R&D Systems, MN, United States) was used. The standard, calibrator, and samples diluted in the dilution buffer PBS-TW, 1.0% Bovine Serum Albumin (Sigma-Aldrich, St. Louis, MO, United States) were applied to the wells and incubated for 60 min at RT with agitation. Following another three washes in PBS-TW, 100 μL of biotinylated mAb 22-21-9 diluted to 1.25 μg/mL in PBS-TW was added to each well. The plates were incubated for 60 min at RT with agitation, followed by three times of washing in PBS-TW and incubated with 100 μL of horseradish peroxidase (HRP)-conjugated streptavidin (Invitrogen, Waltham, MA, United States) diluted 1:5000 in PBS- TW for another 60 min at RT with agitation. Lastly, the plates were washed three times in PBS-TW and incubated with 100 μL TMB-Ultra (Thermo Fisher Scientific) in each well for 10 min at RT in the dark. Addition of 100 μL/well of 0.2 M H_2_SO_4_ stopped the color development and the optical density (OD) was measured at 450 nm. Recorded OD values were used to generate a 9-point standard curve using four-parameter logistic fit model using Softmax Pro® (Molecular Devices, San Jose, CA, United States). The standard, calibrator, and samples were analyzed in duplicates. GraphPad Prism 10.0 (GraphPad Software, Boston, MA, United States) was used for data analysis.

### 2.3 Reference interval of kallistatin in human plasma

The reference interval for kallistatin was established according to the EP28 A3C guideline from the Clinical Laboratory Standards Institute [27]. Citrate stabilized plasma from 100 healthy blood donors (47 women, 52 men, and 1 unknown) and EDTA stabilized plasma from 95 healthy blood donors (45 women, 49 men, 1 unknown) served as reference material.

The plasma protein concentration of kallistatin was assessed as described above. The assay was transferred to a Bio-Plex 200 Multiplex System (Bio-Rad Laboratories, Hercules, CA, United States) immunoassay for the measurement of the reference interval of kallistatin in human citrate and EDTA plasma.

In brief, capture antibody 22-21-7 coupled beads (Bio-Plex Pro magnetic COOH beads, Bio-Rad Laboratories), labelled with two fluorescent dyes for bead identification, were incubated with plasma samples or plasma standard. Binding of kallistatin was detected using biotinylated detection antibody 22-21-9, as described in the previous section. Beads were then incubated with a reporter streptavidin-phycoerythrin (SA-PE) (BD, Franklin Lakes, NJ, USA) conjugate and passed through the Bio-Plex 200 array reader, which measures the fluorescence of the bound SA-PE. A pool of EDTA plasma from six volunteers (three male and three female), with concentrations of kallistatin determined by the ELISA assay, was used as a standard for the standard curve. The protein concentrations in plasma samples were calculated using GraphPad Prism 10.0 (GraphPad Software, Boston, MA, United States). All samples were measured in duplicates and if the CV value for a sample was below 10% the sample was reanalyzed.

### 2.4 Tissue-conditioned media samples from AAA patients and control samples by ELISA

A total of 102 tissue-conditioned media samples from different layers of human AAA and control human abdominal aorta wall were collected from donors at University Paris Diderot and prepared as described in Ref. [28]. Briefly, AAA tissue was dissected into wall (adventitia, n = 20; media, n = 21), ILT (n = 21), and control wall (adventitia, n = 20; media, n = 20), cut into 5 mm^2^ pieces and separately incubated in RPMI-1640 medium containing antibiotics and an antimycotic (Gibco™) for 24 h at 37 °C (6 mL/g of wet tissue). Supernatant was collected by centrifugation (3000 g, 10 min, 20 °C) and frozen at −80 °C. Ethical approval for the human aortic tissues was obtained from the Institutional Review Board (approval number: IRB0000388). Informed consent was obtained for each patient, and the RESAA and AMETHYST protocols were approved by the French ethics committee (CPP Paris-Cochin, approval number: 2095, 1930, and 1931). Control human abdominal aortas were collected from deceased organ donors with the authorization of the French Biomedical Agency (approval number: PFS 09–007, BBMRI network, BB-0033–00029).

### 2.5 Immunohistofluorescence analysis

Formalin-fixed OCT-embedded AAA samples (n = 5) were sectioned in 5 μm thickness, blocked for 30 min at room temperature in 1% BSA in PBS, and incubated overnight at 4 °C with in-house primary antibody against kallistatin (mAb 22-21-7) (10 μg/mL in 1% BSA in PBS). An anti-chicken C3 antibody was used as a negative control (10 μg/mL) [29]. After washing three times in 1% BSA in PBS sections were incubated with secondary antibody Alexa Fluor Plus 555-conjugated goat anti-mouse IgG (Thermo Fisher Scientific, Waltham, MA, United States) (1:300 in 1% BSA in PBS) for 2 h at room temperature. After the sections were washed three times in PBS, coverslips were mounted with SlowFade Gold Antifade Mountant with DAPI (Thermo Fisher Scientific) and scanned by Danish Spatial Imaging Consortium (DanSIC) using High throughput Olympus Slide Scanner VS200 (Olympus Corporation, Tokyo, Japan). Ethical approval was acquired from the Regional Committees on Health Research Ethics for Southern Denmark (OAB, project ID: S-20140202).

### 2.6 Recombinant expression and purification of kallistatin

Kallistatin was transiently expressed in ExpiCHO-S cells (Thermo Fisher Scientific) using pcDNA3.4 Topo plasmid encoding kallistatin amino acid residues 1-427 followed by the sequence GSGSHHHHHHH (Invitrogen). Briefly, ExpiCHO-S cells were cultured in ExpiCHO expression medium (Thermo Fisher Scientific) in a shaker incubator at 125 rpm at 37 °C, and 8.0% CO_2_. The day before transfection, ExpiCHO-S cells were diluted in ExpiCHO expression medium to 3.5 × 10^6^ cells/mL and cells were transfected using ExpiFectamine (Thermo Fisher Scientific) according to the manufacturer's protocol. Before transfection, cells were diluted to 6 × 10^6^ cells/mL in ExpiCHO expression medium and transfected using a total of 1 μg plasmid/mL of ExpiCHO-S cells. One day after transfection, ExpiCHO feed and ExpiCHO enhancer (Thermo Fisher Scientific) were added according to the manufacturer's protocol and cell were transferred to a shaker incubator at 125 rpm, at 32 °C, and 5.0% CO_2_. Five days after transfection, ExpiCHO feed was added according to manufacturer's protocol, and 12 days after transfection, the culture supernatant was harvested by centrifugation at 10.000 x g for 30 min at room temperature.

For purification of recombinant expressed kallistatin, the ExpiCHO supernatant was filtered using 0.22 μM filter (Millipore Corporation, MA, United States). The filtered supernatant was applied to a 5 ml His Trap column (Cytiva, Marlborough, MA, United States) equilibrated in wash buffer (50 mM NaH_2_PO_4_ 300 mM NaCl, 10 mM imidazole pH 8.0) at 2 mL/min, washed with 10 column volumes wash buffer and step eluted with wash buffer supplemented with 300 mM imidazole, all done using an Äkta Pure fast protein liquid chromatography (FPLC) apparatus (Cytiva). The purified protein was buffer exchanged into 10 mM HEPES, 140 mM NaCl, pH 7.4 using a HiTrap desalting column (Cytiva) according to manufacturer's instruction.

### 2.7 Immunoaffinity purification of kallistatin from human plasma

Immunoaffinity purification of kallistatin from human plasma was performed by covalent coupling 2 mg of anti-kallistatin mAb 22-21-7 onto 1 g of cyanogen-bromide (CNBr)-activated Sepharose 4B (GE HealthCare). This matrix was placed in an FPLC column and normal human citrate plasma from individuals without known disease was used as source of kallistatin for the purification. For purification, plasma was diluted 1:2 in PBS, pH 7.4 (Gibco™) and filtered using 0.45 μM filter (Millipore Corporation). The filtered plasma was applied to a 1.5 mL XK16 column (Pharmacia Biotech, GE Healthcare, NY, United States) equilibrated in wash buffer (PBS, pH 7.4) at 1 mL/min, washed with 10 column volumes wash buffer and eluted with 0.5% citric acid directly into Tris, pH 8. Kallistatin was immunoaffinity purified from the plasma using an automated Amersham Biosciences Äkta FPLC system (Amersham Biosciences, GE Healthcare, NY, United States). The purified native kallistatin was dialyzed overnight against PBS at 4 °C in a Spectra/Por® Dialysis Membrane MWCO 6-8 kD (Spectrum Lifesciences, LLC, Rancho Dominguez, CA, United States). The native kallistatin was then depleted of human serum albumin (HSA) and immunoglobulin (Ig) using High-Select™ HSA/Ig Depletion Midi Columns (Thermo Fisher Scientific).

### 2.8 SDS-PAGE

Different samples of kallistatin were subjected to SDS-PAGE using Bolt™ Bis-Tris Plus Mini Protein Gels, 4-12%, 1.0 mm, WedgeWell™ format (Invitrogen) in NuPAGE™ MES SDS Running Buffer (Invitrogen) and using the molecular weight marker Novex™ Sharp Unstained Protein Standard (Invitrogen). Prior to loading samples on the gel, the samples were mixed 1:4 with LDS samples buffer (Invitrogen) and 1:10 with 1 M dithiothreitol for the reduced samples. The gel was run for approximately 50 min at 180 V followed by staining in Coomassie SimplyBlue™ SafeStain (Invitrogen) for 1 h and destained overnight in H_2_O.

### 2.9 ^18^O labelling

Recombinant and native kallistatin were ^18^O labelled by lyophilizing and resuspending 10 μg of recombinant and native kallistatin in 10 μL 95% H_2_^18^O (Sigma-Aldrich) and incubated with 2 μg Peptide-N-Glycosidase F (PNGase F) (Promega, Madison, WI, United States) for 24 h at 37 °C. The samples were subjected to SDS-PAGE and stained in Coomassie as described above, prior to being in-gel digested as described as below.

### 2.10 In-gel digestion

Bands were cut from SDS-gels and cut into ∼2 mm square pieces. The gel pieces were washed in 50 mM NH_4_HCO_3_ for 2 min, washed twice in 100% MeCN for 5 min at room temperature with agitation, and then incubated with 10 mM dithiothreitol for 30 min at 56 °C and cooled to room temperature. This was followed by alkylation with 55 mM iodoacetamide in 100 mM MeCN for 30 min in the dark at room temperature. The reduction and alkylation was followed by another washing step as described above. Protein digestion was achieved by adding 11 ng/μL in-house methylated trypsin in 30 μL 50 mM NH_4_HCO_3_ to each sample. The gel pieces were left on ice for 20 min. Excess liquid was removed, 50 μL of 50 mM NH_4_HCO_3_ was added, and the samples incubated at 37 °C overnight.

### 2.11 LC-MS/MS

The digested samples were prepared for LC-MS/MS by micro-purification on custom made micro columns. The columns were made in GELoader tips (Eppendorf, Hamburg, Germany) using a small piece of Empore C18 disk (3 M, Saint Paul, MN, United States) as a plug. Two μL of POROS R2 resin material (Thermo Fischer Scientific) with 20 μL 100% MeCN was loaded onto the columns. The columns equilibrated in 0.1% trifluoroacetic acid (TFA) prior to loading the samples. After loading the samples, the columns were washed twice in 0.1% TFA and then eluted with 70% MeCN, 0.1% TFA solution. The samples were analyzed by LC-MS/MS using an EASY-nLC 1200 (Proxeon, Odense, Denmark) equipped with a 150 mm × 75 μm ID pulled-emitter column, packed with 3 μm Reprosil Pur C18 material (Dr. Maisch, Germany), operated at 250 nL/min coupled to an Exploris 480 mass spectrometer (Thermo Fischer Scientific), using a 1-h gradient. Data were collected at an MS1 resolution of 120000 and an HCD collision energy setting of 30% for the deglycosylated samples and a stepped collision HCD energy of 20% and 25% was used for the native in order to facilitate the detection of glycosylated samples [30]. The MS2 data was collected with a resolution of 15000.

### 2.12 Kallistatin deglycosylation with PNGase F and thermic polymerization

For thermic polymerization, samples of 10 μg recombinant expressed kallistatin and 10 μg native kallistatin were incubated with 2 μL PNGase F (Promega) for 24 h at 37 °C. Samples of recombinant expressed kallistatin, native kallistatin, deglycosylated recombinant expressed kallistatin, and deglycosylated native kallistatin were thermically polymerized by heating at 63 °C with agitation for 4 h. For mass spectrometric analysis a 10 μg sample and 0.2 μg trypsin in 50 mM phosphate buffer, pH 7.5, were each lyophilized and redissolved in 98% ^18^O-water (CogecNet, Les Ulis, France) prior to being mixed and incubated overnight at 37 °C. All the samples were then subjected to SDS-PAGE and stained by Coomassie as described above. In a separate experiment an additional 2 μl PNGase A (Promega) was added to recombinant expressed kallistatin before incubation.

### 2.13 Identification of glycopeptidess

The RAW files from the mass analysis were converted to mgf files using msConvertGUI [31]. The mgf files were then loaded into GPMAW version 14.2 (Lighthouse data, Odense, Denmark) and non-glycosylated peptides were analyzed with the MS/MS search function while glycopeptides were analyzed with the Find glyco functions. Both searches were carried out with an ms precision of 10 ppm and an ms/ms precision of 0.1 Da. While the ms/ms search uses an external search engine (X!Tandem [32]) the glycopeptide search uses an internal database of approximately 300 glycans, performs an initial mass search and then validates the deconvoluted spectra based on 1) carbohydrate oxonium ions 2) fragments of the peptide + carbohydrates in the core. Hits can easily be viewed and manually validated in the built-in spectrum viewer which also marks all glycan fragments (Suppl. Fig. S1). Deglycosylation was identified as a mass increase on asparagine of 2.988 Da arising from deamidation (Asn-Asp) and the substitution of oxygen for 18O (Suppl. Fig. S2).

### 2.14 Functionality of kallistatin

The functional impact of kallistatin's inhibitory effect on tissue kallikrein activity was analyzed using the tissue kallikrein sensitive fluorogenic substrate (H-Pro-Phe-Arg-AMC) (Bachem Holding AG, Bubendorf, Switzerland). A buffer of 50 mM Tris, 150 mM NaCl, pH 7.4 prewarmed to 37 °C was used throughout the experiment. A total concentration of 10 μg/mL of recombinant kallistatin, 10 μg/mL of kallistatin treated with PNGaseF (1 unit of 500 units/mL) or 10 μg/mL thermic polymerized kallistatin were incubated with a total concentration of 10 μg/mL recombinant tissue kallikrein for 2 h at 37 °C with agitation. A volume of 100 μL of each sample was added to a Nunc™ 96-Well Polystyrene Round Bottom Plate (Thermo Fischer Scientific), and 20 μL (100 mM) of the tissue kallikrein sensitive fluorogenic substrate was added to each sample. The experiments were followed for 2 h and 10 min at 37 °C with agitation and Relative Fluorescence Units (RFU) recordings (excitation wavelength 355 nm, emission wavelength 460 nm) every 12 s on a Thermo Scientific Fluoroskan Microplate Fluorometer Reader (Thermo Fisher Scientific).

### 2.15 Statistical analysis

The kallistatin glycosylations were analyzed using the X!Tandem search engine through GPMAW version 14.2 (Lighthouse data, Odense, Denmark). A program was written in Python to calculate the relative percentage of deglycosylation for each potential glycosylation site. The program takes the output from an X!Tandem search, identifies the spectra of all peptides for each of the potential glycosylation sites and calculates the fraction containing a 3 Da increase in mass on asparagine residues. A sum of intensities is then calculated and used to compute the relative percentage of glycosylation for each of the glycosylation sites.

To explore potential differences in contact system protein levels in tissue-conditioned media samples of different layers of the aortic wall between individuals with and without AAA, Kruskal-Wallis test followed by Dunn's multiple comparisons test were conducted. These results were visualized with scatter dot plots with individual datapoints and median with IQR per group stratified by aneurysm status. The plots were created using GraphPad Prism 10.0.

## 3 Results

### 3.1 Human kallistatin specific mAbs for establishment of kallistatin specific sandwich ELISA

**Fig. 1:**
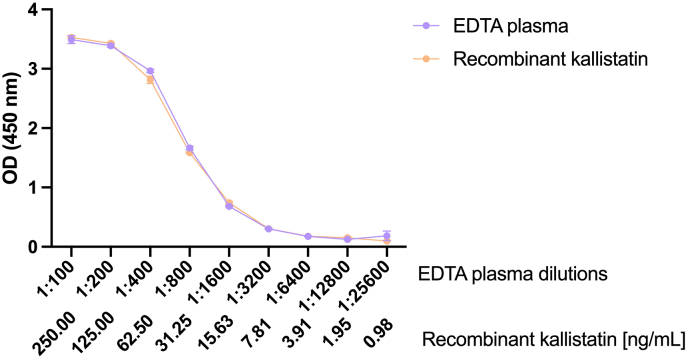
Human kallistatin specific non-competitive sandwich ELISA. Measurements of kallistatin levels by kallistatin specific non-competitive Enzyme-Linked Immunosorbent Assay (ELISA) using anti-kallistatin monoclonal antibody (mAb) 22-21-7 as capture antibody and biotinylated anti-kallistatin mAb 22-21-9 as detection antibody. Kallistatin levels were measured in human EDTA plasma and recombinant kallistatin in twofold dilutions. The kallistatin signals are given as optical density (OD) at 450 nm. Error bars indicate median and range of a double determination.

A panel of kallistatin-specific mAbs, initially screened against recombinantly expressed kallistatin, was generated (data not shown). These mAbs were evaluated in various capture-detection combinations, resulting in the establishment of a kallistatin-specific non-competitive sandwich ELISA using anti-kallistatin mAb 22-21-7 as capture antibody and biotinylated anti-kallistatin mAb 22-21-9 as detection antibody. Signals of kallistatin in EDTA plasma and recombinant kallistatin were obtained (Fig. 1).

### 3.2 Reference interval of kallistatin in human citrate and EDTA plasma

**Fig. 2:**
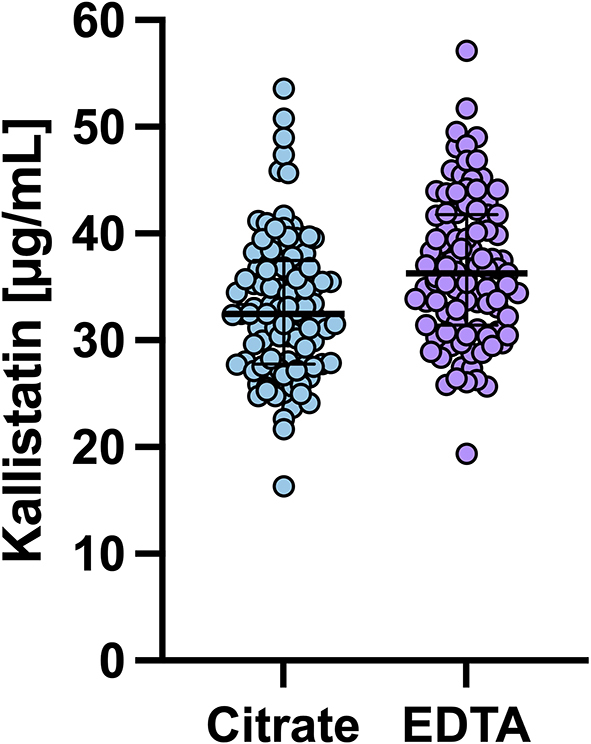
Human kallistatin levels in citrate and EDTA plasma. Kallistatin levels determined in citrate and EDTA stabilized plasma from a reference population of 100 healthy blood donors (47 women, 52 men, 1 unknown) and 95 healthy blood donors (45 women, 49 men, 1 unknown), respectively. The median plasma level of kallistatin in citrate was 32.50 (27.78 – 37.48) μg/mL and 36.26 (31.44-41.78) μg/mL in EDTA. The median level of kallistatin in EDTA plasma was significantly higher compared to citrate plasma, p = 0.0002. Data are presented as individual data points and medians with interquartile range (IQR 25-75%) per group. Data were analyzed by using unpaired *t*-test.

The kallistatin specific ELISA setup was transferred to a Bio-Plex 200 Multiplex System (Bio-Rad) immunoassay to measure a reference interval of kallistatin in human citrate plasma (n = 100; 47 women, 52 men, 1 unknown) and EDTA plasma (n = 95; 45 women, 49 men, 1 unknown). A scatter dot plot with individual data points and median with interquartile range (IQR) 25-75% display the distribution of kallistatin in citrate and EDTA plasma (Fig. 2).

The citrate plasma level of kallistatin was normally distributed (*p* > 0.1000). The median level of kallistatin was not significantly different between women 32.38 (27.55 – 39.17) μg/mL and men 32.59 (29.39 – 36.71) μg/mL, *p* = 0.8533. Hence, results for citrate plasma were combined establishing a reference interval of 27.78 – 37.48 with a median level of 32.50 μg/mL. The EDTA plasma level of kallistatin was normally distributed (*p* > 0.1000). The median level of kallistatin in EDTA plasma was not significantly different between women 35.33 (30.38 – 41.84) μg/mL and men 37.45 (32.26 – 42.24) μg/mL, *p* = 0.3266. Hence, results for EDTA plasma were combined establishing a reference interval of 31.44 – 41.78 with a median level of 36.26 μg/mL. We observed a significantly higher median level of kallistatin in EDTA plasma (36.26 μg/mL) compared with citrate plasma (32.50 μg/mL), *p* = 0.0002.

### 3.3 Evaluation of kallistatin in abdominal aortic aneurysm tissue samples

**Fig. 3:**
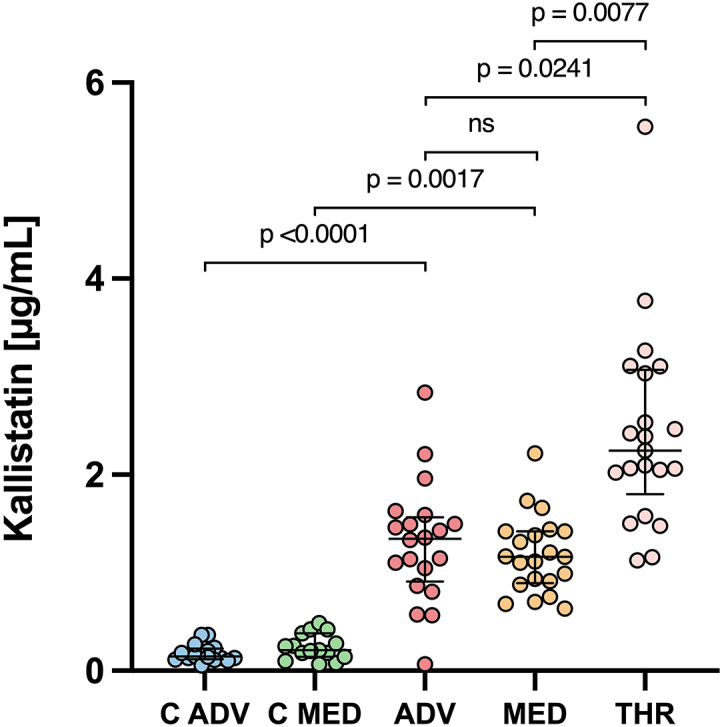
Measures of kallistatin in aortic wall-tissue supernatant from patients with AAA compared with control samples. Concentrations of kallistatin were measured by enzyme-linked immunosorbent assay (ELISA) in tissue-conditioned media of the different layers of human abdominal aortic aneurysm (AAA): Tunica media (MED, n = 21), tunica adventitia (ADV, n = 20), and intraluminal thrombus (THR, n = 21), and in different layers of control human abdominal aorta wall: control tunica media (C MED, n = 20) and control tunica adventitia (C ADV, n = 20). Data are presented as individual data points and medians with interquartile range (IQR 25-75%) per group. Data were analyzed by Kruskal-Wallis test followed by Dunn's multiple comparisons test. ns, not significant.

Scatter dot plots with individual data points and median with IQR 25-75% visualize the distribution for kallistatin in tissue-conditioned media samples (Fig. 3). The levels of kallistatin were analyzed in tissue-conditioned media of different layers of human AAA: media (MED, n = 21), adventitia (ADV, n = 20), and thrombus (THR, n = 21), and different layers of control human abdominal aorta wall: media (C MED, n = 20) and adventitia (C ADV n = 20) by ELISA.

We observed significantly higher levels in the medial layer of AAA MED compared with control aorta (1.16 μg/mL [IQR: 0.90-1.42] vs. 0.21 μg/mL [IQR: 0.14-0.38], *p* = 0.0017), and a higher level in the adventitial layer of AAA compared with their control (1.35 μg/mL [IQR: 0.91-1.6] vs. 0.14 μg/mL [IQR: 0.12-0.23], *p* < 0.0001). When comparing the kallistatin levels between the different AAA layers, we found a higher level in AAA thrombus compared with AAA media (2.25 μg/mL [IQR: 1.8-3.1] vs. 1.16 μg/mL), *p* = 0.0077) or AAA adventitia (2.25 μg/mL vs. 1.35 μg/mL), *p* = 0,0241). No significant difference between the AAA MED and AAA ADV was observed (Fig. 3).

### 3.4 Presence of kallistatin in abdominal aortic aneurysm tissue

**Fig. 4:**
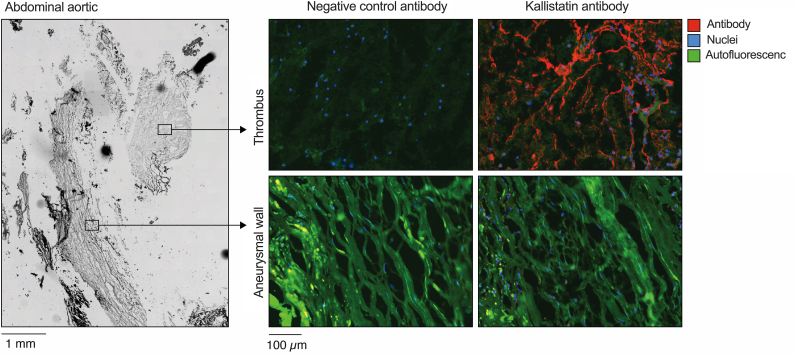
Immunohistofluorescence analysis of kallistatin in abdominal aortic aneurysms. Detection of kallistatin in the aneurysmal wall of all patients (n = 5). In samples containing thrombotic material, kallistatin was intensely localized within the thrombus (top row).

The observed pattern of kallistatin in tissue-conditioned media samples was supported by an immunofluorescence analysis of AAA lesions. We found kallistatin was detectable within the vascular tissue with a particularly stronger signal in the intraluminal thrombus (Fig. 4).

### 3.5 Recombinant and native kallistatin

**Fig. 5:**
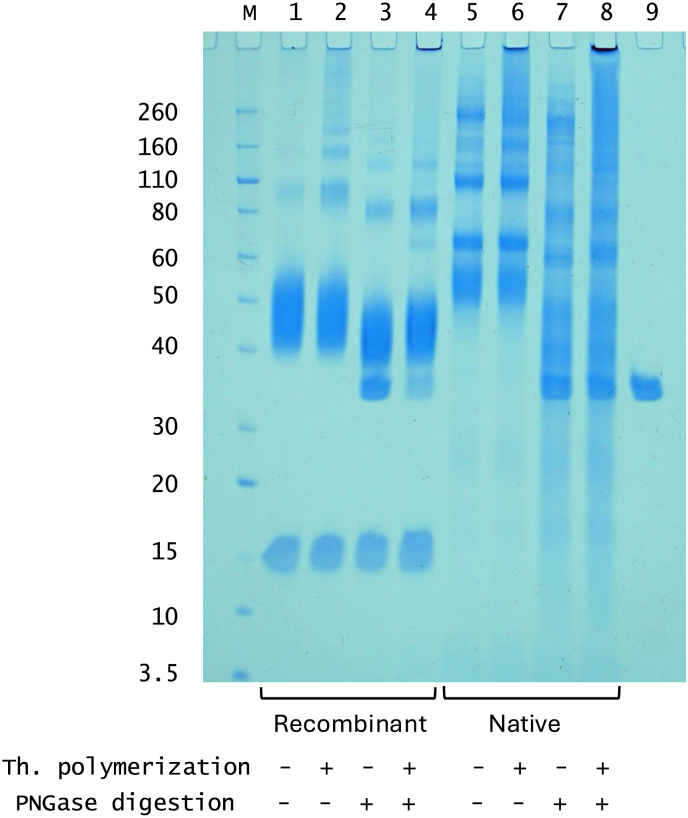
Glycosylation and polymerization of recombinant and native kallistatin purified from human plasma. Recombinant (lanes 1-4) and native (lanes 5-8) kallistatin were treated with thermic polymerization and/or deglycosylation by Peptide-N-Glycosidase F (PNGase F) as indicated in the legend prior to being run on a non-reduced SDS-PAGE and Coomassie-stained. M indicates the molecular weight marker, P is PNGaseF loaded at similar concentration as in the assay.

Native and recombinant kallistatin, were subjected to SDS-PAGE followed by Coomassie SimplyBlue™ SafeStain (Fig. 5). Under non-reducing conditions, recombinant kallistatin showed as a ∼14 kDa wide band centered around 48 kDa, while native kallistatin showed a narrower band of ∼8 kDa centered slightly higher around 55 kDa (Fig. 5, lane 1 and 5). Polymerized kallistatin was observed in the wells of the gel, see below. Upon reduction, the bands compacted slightly but showed the same characteristics (Suppl. Fig. S3, lane 1 and 5). These results led us to evaluation and characterization of kallistatin by mass spectrometry.

### 3.6 Effect of glycosylation levels in kallistatin on polymerization

Recombinant and native kallistatin were treated with PNGase F for deglycosylation followed by a thermic polymerization. In this study, the term *polymerization* refers to heat-induced, disulfide-linked multimer formation observed under non-reducing SDS-PAGE conditions, which collapses to monomer under reducing conditions. The different combinations of PNGase F and thermic polymerization of kallistatin were subjected to SDS-PAGE analysis followed by Coomassie staining (Fig. 5).

For recombinant kallistatin we observed a minor reduction in molecular weight upon treatment with PNGase F. The reason for only a partial glycan removal is most likely caused by core focusylation [33,34], while native kallistatin was mostly deglycosylated. Incubating the recombinant kallistatin with both PNGase F and PNGase A still left a small part of glycosylation (Suppl. Fig. S4).

Upon thermic polymerization (Fig. 5, lanes 4 and 8) a strong band was observed in the well of the gel for both recombinant and native kallistatin after PNGase treatment indicating a high degree of polymerization. The presence of kallistatin in the wells was verified by mass spectrometry. Weaker bands in the wells of lane 2 and 6 indicate that kallistatin oligomerizes to a small degree even under native conditions when subjected to thermic polymerization, but the oligomerizations strongly increased after deglycosylation (Fig. 5 lane 2 vs 4 and lane 6 vs 8). Thermic polymerization of the PNGase F and A treated sample showed complete polymerization of the lowest band having the least glycosylation level.

Performing the experiment under reducing conditions showed that the multimers collapses to a monomer (Suppl. Fig. S3), indicating that the polymerization is caused by disulfide interchange, rather than irreversible precipitation.

### 3.7 Mass spectrometry-based characterization of kallistatin glycosylation sites

Native and recombinant kallistatin were treated with PNGase F for deglycosylation in ^18^O labelled water. This allows for identification and quantitation of glycosylations by measuring the relative intensity of the non-glycosylated (Asn containing) peptide with the deglycosylated (^18^O modified Asp) peptide, by a mass difference of 3 Da [35]. Samples were subjected to SDS-PAGE and stained with Coomassie. While native kallistatin was almost completely deglycosylated (Fig. 5, lane 7) only a smaller part of recombinant kallistatin was deglycosylated (Fig. 5, lane 3). This is likely caused by the innermost GlcNAc in the glycan being focusylated which inhibits PNGaseF. Both kallistatin bands in the 45-55 kDa region were cut into three bands: top, middle, and bottom, and all six bands were in-gel digested and analyzed by mass spectrometry. All bands showed clear presence of both the non-glycosylated and the deglycosylated (+3 Da) peptide for all four potential glycosylation sites (Asn 33, Asn 108, Asn 157 and Asn 238). The relative distribution of the deglycosylated peptides was determined by the ratio of the intensities of the non- and de-glycosylated peptides quantified using an in-house program and is shown in Suppl. Fig. S5. The ratios show very little deviation in the observed relative occupancy of the four glycosylation sites for the native kallistatin, while relatively large differences can be observed for the recombinant kallistatin. This indicates that upon PNGase F digestion glycans in the native protein are removed from the four glycan sites in an equal ratio, while the recombinant protein showed removal of glycans from sites Asn 33 and Asn 108 to a much higher degree than Asn 57 indicating differences in glycan composition. However, it does shows that for both kallistatins all four potential glycosylation sites are glycosylated.

### 3.8 Mass spectrometry-based characterization of kallistatin glycans

**Fig. 6:**
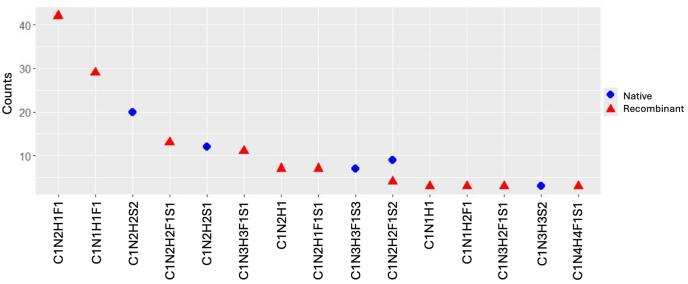
Glycan composition of native and recombinant kallistatin. Plot of observed glycan compositions against number of identified glycans of the given composition. Blue circles represent glycans from native kallistatin while red triangles represent glycans from recombinant kallistatin. Nomenclature used: C: glycan core (2 x N-acetylglucosamine, 3 x Hexose); N: N-acetylglucosamine; H: Hexose; F: Fucose; S: Sialic acid.

For structural analysis of the N-linked glycans, bands of intact kallistatin from the SDS-gel separation were cut and in-gel tryptic digested. Samples were micro-purified and analyzed by LC-MS/MS. The ‘Locate glycan’ function in GPMAW 14.2 was used to identify glycans and all spectra of glycopeptides were manually verified. More than 150 spectra were identified having 15 different N-linked glycan structures, each identified with at least three spectra (Fig. 6).

While five different glycans were found for native kallistatin, eleven were found for recombinant kallistatin, and only a single glycan structure was shared between native and recombinant kallistatin. A characteristic feature was that the majority of glycans for the recombinant kallistatin were focusylated, while this was only the case for two, relatively low abundant, of the native kallistatin glycans (Fig. 6).

### 3.9 Functional effect of deglycosylated and polymerized kallistatin on tissue kallikrein activity

**Fig. 7:**
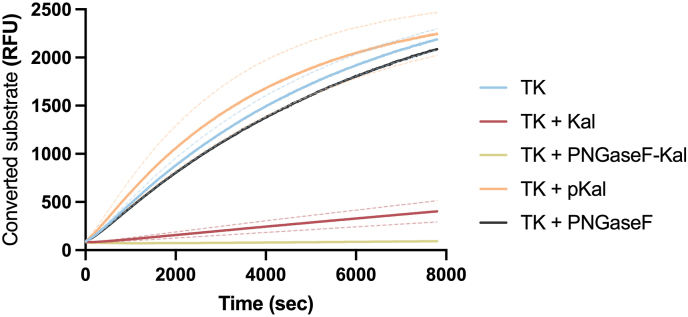
Analysis of kallistatin functionality on tissue kallikrein. The functional effects of different forms of recombinant kallistatin on tissue kallikrein (TK): kallistatin (Kal), kallistatin treated with PNGase F (PNGaseF-Kal), and thermic polymerized kallistatin generated by incubation at 63 °C for 4 h (pKal)—were assessed using a fluorogenic tissue kallikrein substrate. Relative Fluorescence Units (RFU) are plotted against time (seconds). Tissue kallikrein (10 μg/mL) was incubated with either buffer (blue), kallistatin (red), PNGaseF-treated kallistatin (green), polymerized kallistatin (orange), or PNGaseF only (black). Error lines (dashed lines) indicate median and range of two independent experiments.

Recombinant kallistatin, either untreated, PNGaseF treated, or polymerized, was incubated with tissue kallikrein and a tissue kallikrein sensitive fluorogenic substrate (Fig. 7). Tissue kallikrein alone showed activity over time (Fig. 7, blue TK) and as expected, co-incubation with recombinant kallistatin resulted in inhibition of tissue kallikrein activity. (Fig. 7, red TK + Kal). Polymerized kallistatin lost its inhibitory capacity, showing no effect on tissue kallikrein activity (Fig. 7, orange TK + pKal). PNGaseF treated kallistatin retained its inhibitory function (Fig. 7, green TK + PNGaseF-Kal). The control with PNGaseF only showed minimal effect on tissue kallikrein activity, confirming that the observed effects are attributable to kallistatin modification.

## 4 Discussion

In this study, we developed a panel of mAbs specifically binding kallistatin, which enabled the establishment of a kallistatin-specific sandwich ELISA. These antibodies facilitated both quantitative and qualitative analyses, allowing detailed characterization of kallistatin's, glycosylation profile and localization in vascular tissue.

We used the established assay to determine the reference interval for kallistatin in citrate and EDTA stabilized plasma. The reference interval is given as IQR 25-75% of the distribution corresponding to 27.78 – 37.48 μg/mL (median of 32.50 μg/mL) for citrate plasma and 31.44 – 41.78 μg/mL (median 36.26 μg/mL) for EDTA plasma. These data demonstrate higher kallistatin levels in EDTA plasma. Previous studies have reported kallistatin concentrations in human plasma, but with varying cohort sizes and demographic compositions. For example, Chao et al. reported levels of 22.1 μg/mL in 30 healthy individuals using citrate plasma [2], while Zhu et al. reported levels of 27.9 μg/mL in males and 26.8 μg/mL in females (mean (SD)) in 318 apparently healthy African American adolescents (aged 14–19 years, 48.1% females) [36]. The present data add comparative information on kallistatin levels in both citrate and EDTA plasma within a healthy adult population and may support future efforts in assay standardization and interpretation across different sample types.

To explore the presence of kallistatin in the local vascular environment, we applied the developed assay to tissue-conditioned media from different layers of the abdominal aorta in patients with AAA and controls. We found that the kallistatin levels differed between control and AAA samples, with elevated levels in AAA wall layers and particularly higher levels in the intraluminal thrombus compared to non-aneurysmal tissue controls. These results not only suggest a potential attempt of kallistatin modulating local contact system activity, and thereby a potential role of kallistatin in AAA pathophysiology but also demonstrate that the assay is suitable for assessing kallistatin in both plasma and tissue-conditioned media. Additionally, we showed that a generated kallistatin mAb was suitable for immunohistofluorescence analysis, demonstrating that kallistatin was detectable in aneurysmal tissue and enriched in the intraluminal thrombus.

By generating our own mAbs, we were able to produce stable, large-scale batches suitable for matrix coupling and immunoaffinity purification, which provided native kallistatin in a purity compatible with the structural analyses performed here—an application not supported by standard quantitative assay reagents. Kallistatin was subsequently characterized using both the native and recombinant kallistatin preperations. The native kallistatin was compared with recombinant kallistatin and analyzed by SDS-PAGE followed by Coomassie staining. These results revealed a difference in molecular weight between the native and recombinant kallistatin, showing a molecular weight of 40-54 kDa for recombinant kallistatin and a molecular weight of ∼50-58 kDa for native kallistatin. In the purified native kallistatin fraction, we observe pronounced polymerization, which is most likely attributable to the immunoaffinity-purification procedure. The results indicate a difference in glycosylation levels between the recombinant and native kallistatin, as the production of recombinant proteins is known to involve structural modifications, including alterations in glycosylation profiles [[37], [38], [39]]. Characterizing the glycosylation sites of both native and recombinant kallistatin by mass spectrometry, we established the presence of glycosylation sites on Asn 33, Asn 108, Asn 157, and Asn 238 in both protein forms. The three sites Asn 108, Asn 157 and Asn 238 have previously been described [[8], [9], [10]], while the last site, Asn 33, has only been proposed based on sequence analysis prediction [40]. Our data therefore provide experimental confirmation of the putative glycosylation at Asn 33, which to our knowledge has not previously been demonstrated. While a variety of glycans could be determined on three of the sites, no glycosylated peptides were found for Asn 33. This can be explained by the peptides generated being quite long and having multiple His residues resulting in large and highly charged peptides. While deglycosylation by PNGase F was mostly successful for the native kallistatin, the recombinant kallistatin was deglycosylated to a much lesser degree. Even treatment with a combination of PNGase F and PNGase A left a large part of the recombinant kallistatin glycosylated. The main difference between the two kallistatins is that the glycans attached to the native is relatively simple with only two fucosylated species while the recombinant is considerably more complex and show mostly fucosylated species (Fig. 6). Treatment of a tryptic digest of recombinant kallistatin with PNGase F revealed no presence of glycopeptides when analyzed by mass spectrometry (results not shown) which showed that the core focusylation effectively shielded the glycans on the intact protein from cleavage by PNGase F.

Serpins are known to undergo polymerization under certain conditions, a structural transformation that can significantly affect their biological function and has been implicated in various disease mechanisms. Given kallistatin's classification as a serpin, we investigated its potential to polymerize and the influence of glycosylation on this process. We analyzed its polymerization behavior using SDS-PAGE followed by Coomassie staining. The band patterns revealed that both recombinant and native kallistatin are capable of forming polymers, confirming kallistatin's susceptibility to structural rearrangement. We further explored the influence of glycosylation on polymerization and found that both kallistatins exhibit a higher degree of polymer formation when deglycosylated, although we observed that only a smaller part of recombinant kallistatin was deglycosylated. Our findings thereby concur with studies concerning the critical role of glycosylation in protein folding, stability, solubility, and prevention of protein aggregation [[11], [12], [13]]. Heating kallistatin under reducing condition abolishes the multimerization showing that it is likely caused by creation of random disulfides. Kallistatin has three cysteines, neither participating in disulfide bonding, with two surface-exposed and one slightly hidden (Cys 263) (Suppl. Fig. S6). The four glycosylation sites in kallistatin are located relatively close to the exposed cysteines indicating that one purpose could be to shield the cysteines and prevent multimerization under normal conditions. Upon thermal denaturation the protein unfolds, and the cysteines will be able to form disulfide bonds and thus multimerize. Because only a fraction of kallistatin formed multimers, and these disappeared under reducing conditions, we do not infer a specific serpin polymer mechanism. Instead, we interpret the findings as an increased tendency towards heat-induced, disulfide linked multimer formation, especially after deglycosylation. As the deglycosylation in ^18^O water clearly shows that all four glycosylation sites are only partially glycosylated, there is the possibility of multimers even under native conditions. A weak band of dimeric native kallistatin can be observed on non-reduced gels which is not observed for recombinant kallistatin, most likely due to the increased glycosylation of the recombinant protein.

The functional effect of polymerized and deglycosylated kallistatin on tissue kallikrein demonstrates that polymerization completely abolishes the inhibitory capacity of kallistatin, whereas deglycosylation has no impact on inhibition. While glycosylation does not directly affect inhibitory activity, the observation that deglycosylated kallistatin is more prone to polymerization suggests that glycosylation primarily contributes to structural stabilization rather than functional modulation.

Our findings highlight the potential importance of polymerized kallistatin in disease, as previous studies have shown associations of polymer formation of the serpins, α_1_-antitrypsin, neuroserpin, and C1 inhibitor with the clinical conditions, cirrhosis [20], familial hereditary dementia [18], and hereditary angioedema [19], respectively. Several studies have found kallistatin to play a role in diseases, such as AAA [25,41], diabetic nephropathy [42], and renal fibrosis [43]. The role of polymerized kallistatin should therefore be considered in the context of different diseases and biological processes.

In conclusion, we have developed a kallistatin-specific ELISA that enables reliable quantification of kallistatin in both plasma and tissue-conditioned media. While similar assays exist, our approach allowed for broader application and facilitated detailed structural analysis of native kallistatin from human plasma. Through immunoaffinity purification and mass spectrometry, we confirmed the presence of four glycosylation sites, including experimental validation of the previously predicted site at Asn 33, adding new evidence to kallistatin's glycosylation profile.

Furthermore, we demonstrated that kallistatin is capable of polymerization, particularly when deglycosylated, which abolishes its inhibitory activity against tissue kallikrein. These findings suggest that glycosylation plays a stabilizing role in kallistatin structure rather than directly modulating its inhibitory function.

Overall, our study provides new insights into the structural properties of kallistatin, including its glycosylation and polymerization behavior, and offers a foundation for future investigations into its role in disease.

## Declarations of generative AI use

During the preparation of this work the authors used ChatGPT (Open AI) to improve language, grammar, and clarity. After using this tool/service, the authors reviewed the content and take full responsibility for the content of the publication.

## Funding sources

This work was supported by research grants from the 10.13039/501100009708Novo Nordisk Foundation, grant number NNF22OC0079471. This work was also supported by A.P. Møller foundation, grant number L-2022-00136.

## CRediT authorship contribution statement

**Stephanie T.D. Pham:** Conceptualization, Data curation, Formal analysis, Funding acquisition, Investigation, Methodology, Project administration, Validation, Visualization, Writing – original draft, Writing – review & editing. **Kristian W. Nielsen:** Data curation, Formal analysis, Investigation, Software, Validation, Visualization, Writing – review & editing. **Jonas H. Graversen:** Investigation, Methodology, Writing – review & editing. **Nanna Kristensen:** Investigation, Methodology, Writing – review & editing. **Lasse B. Steffensen:** Investigation, Methodology, Validation, Visualization, Writing – review & editing. **Andrea R. Lundgaard:** Investigation, Methodology, Writing – review & editing. **José L. Martín-Ventura:** Methodology, Resources, Writing – review & editing. **Peter Højrup:** Conceptualization, Data curation, Formal analysis, Project administration, Resources, Software, Supervision, Visualization, Writing – original draft, Writing – review & editing. **Yaseelan Palarasah:** Conceptualization, Funding acquisition, Project administration, Resources, Supervision, Validation, Visualization, Writing – original draft, Writing – review & editing.

## Declaration of competing interest

The authors state that they have no conflict of interest.

## Data Availability

Data will be made available on request.
