# Supplementary material for: Structural and functional characterization of human kallistatin
Source: Biochem Biophys Rep. 2026 Jun 2;46:102651. doi: 10.1016/j.bbrep.2026.102651 (PMC13251767; doi:10.1016/j.bbrep.2026.102651)
Supplement: Multimedia component 1 [file mmc1.docx]

**SUPPLEMENTAL MATERIAL**

Structural and Functional Characterization of Human Kallistatin

Authors

**Stephanie T. D. Pham^1*^**, Kristian W. Nielsen^2^, Jonas H. Graversen^1^, Nanna Kristensen^2^, Lasse B. Steffensen^3^, Andrea R. Lundgaard^3^, José L. Martín-Ventura^4,5^, Peter Højrup^2^, Yaseelan Palarasah^1^.

**^1^**Inflammation Research Unit, Department of Molecular Medicine, University of Southern Denmark, Odense, Denmark.

**^2^**Department of Biochemistry and Molecular Biology. University of Southern Denmark, Odense, Denmark.

**^3^**Cardiovascular and Renal Research Unit, Department of Molecular Medicine, University of Southern Denmark, Odense, Denmark.

**^4^**Vascular Research Laboratory, IIS-Fundación Jiménez Díaz, Madrid, Spain.

**^5^**CIBER de Enfermedades Cardiovasculares (CIBERCV), Madrid, Spain.

*Corresponding author

Stephanie Thuy Duong Pham

ORCID-ID: 0000-0003-3083-5976

Inflammation Research Unit, Department of Molecular Medicine, University of Southern Denmark, Odense, Denmark.

Mail: spham@health.sdu.dk

Phone: +45 40546924

Table of Content

[Supplemental Figure S1: Mass spectra of identified glycan structures 3](#_Toc227054462)

[Supplemental Figure S2: Mass spectra validating Asn33 deglycosylation 11](#_Toc227054463)

[Supplemental Figure S3: Glycosylation and polymerization of recombinant and purified kallistatin from human plasma reduced 12](#_Toc227054464)

[Supplemental Figure S4: Glycosylation and polymerization of recombinant kallistatin with PNGaseF and PNGaseA digestion 13](#_Toc227054465)

[Supplemental Figure S5: The relative distribution of deglycosylated peptides on purified and recombinant kallistatin 14](#_Toc227054466)

[Supplemental Figure S6: 3D structure of kallistatin 15](#_Toc227054467)

# **Supplemental Figure S1:** Mass spectra of identified glycan structures

Representative ms/ms scans of the identified peptide glycan structures. Spectra are headed by peptide sequence + glycan composition.

Peaks recognized as fragment ions are colored according to identified fragment type. Blue colored peaks are oxonium ions, lime colored are Y-ions, typically starting with Peptide + HexNAc with fragments to Peptide + core + HexNAc.

Abbreviations: Cor – N-linked glycan core structure (2 * HexNAc + 3 * Hex); HexNAc – N-acetyl hexosamine; Hex – hexose; Sia – sialic acid.

FLNDTMAVYEAK - (Cor)1 (HexNAc)2 (Hex)2 (Fuc)1 (Sia)1

DFYVDENTTVR - (Cor)1 (HexNAc)1 (Hex)1 (Fuc)1

FLNDTMAVYEAK -(Cor)1 (HexNAc)4 (Hex)4 (Fuc)1 (Sia)1

FLNDTMAVYEAK - (Cor)1 (HexNAc)3 (Hex)2 (Fuc)1 (Sia)1

SQILEGLGFNLTELSESDVHR - (Cor)1 (HexNAc)2 (Hex)2 (Sia)2

DFYVDENTTVR - (Cor)1 (HexNAc)2 (Hex)2 (Sia)1

SQILEGLGFNLTELSESDVHR - (Cor)1 (HexNAc)2 (Hex)1

SQILEGLGFNLTELSESDVHR - (Cor)1 (HexNAc)2 (Hex)1 (Fuc)1

SQILEGLGFNLTELSESDVHR - (Cor)1 (HexNAc)3 (Hex)3 (Sia)2

SQILEGLGFNLTELSESDVHR - (Cor)1 (HexNAc)2 (Hex)2 (Fuc)1 (Sia)2

SQILEGLGFNLTELSESDVHR - (Cor)1 (HexNAc)3 (Hex)3 (Fuc)1 (Sia)1

SQILEGLGFNLTELSESDVHR - (Cor)1 (HexNAc)2 (Hex)1 (Fuc)1 (Sia)1

FLNDTMAVYEAK - (Cor)1 (HexNAc)1 (Hex)1

SQILEGLGFNLTELSESDVHR - (Cor)1 (HexNAc)3 (Hex)3 (Fuc)1 (Sia)3

# **Supplemental Figure S2:** Mass spectra validating Asn33 deglycosylation

**A)**

**B)**

**Fig. S2: Mass spectra validating Asn 33 deglycosylation.** Mass spectra of the N-terminal tryptic peptide 21-50 (pre-kallistatin numbering) containing Asn33 as **A)** deglycosylated in ^18^O water **B)** non-glycosylated. The peptide sequence along with coverage is presented in the top left of each spectrum. The N-terminus is deamidated (pyroglutamic acid) and Cys 31 is carbamidomethylated. b-ions are colored blue and y-ions red. Ions marked with a * are doubly charged. Mass values shown in a green box show a +3 Da increase in mass from non-glycosylated to deglycosylated peptide representing deamidation (Asn-Asp) and the addition of an ^18^O atom. The mass values between the two spectra show a difference of 1.5 Th as the fragments are doubly charged.

# **Supplemental Figure S3:** Glycosylation and polymerization of recombinant and purified kallistatin from human plasma reduced

**Fig. S1:** **Glycosylation and polymerization of recombinant and purified kallistatin from human plasma reduced.** Recombinant (lanes 1-4) and purified (lanes 5-8) kallistatin were treated with thermic polymerization (th. polymerization) and/or deglycosylation by Peptide-N-Glycosidase F (PNGase F) as indicated in the legend prior to being run on a reduced SDS-PAGE and Coomassie-stained. M indicates the molecular weight marker, P indicates the PNGaseF marker.

# **Supplemental Figure S4:** Glycosylation and polymerization of recombinant kallistatin with PNGaseF and PNGaseA digestion

**Fig. S2:** **Glycosylation and polymerization of recombinant kallistatin with PNGaseF and PNGaseA digestion.** Recombinant kallistatin were treated with thermic polymerization (th. polymerization) and/or deglycosylation by Peptide-N-Glycosidase (PNGase) F and/or PNGase A, as indicated in the legend, prior analysis by SDS-PAGE followed by Coomassie-staining. M indicates the molecular weight marker, P indicates the PNGaseF marker.

# **Supplemental Figure S5:** The relative distribution of deglycosylated peptides on purified and recombinant kallistatin

**Fig. S3: The relative distribution of deglycosylated peptides on purified and recombinant kallistatin.** Bar plot of relative glycosylation percentage of each glycosylation site for purified (left) and recombinant kallistatin (right). Samples of ^18^O labelled purified and recombinant kallistatin were subjected to Sodium Dodecyl Sulfate-Polyacrylamide Gel Electrophoresis (SDS-PAGE) and staining with Coomassie. Both purified and recombinant kallistatin bands in 45–55 kDa region were separated into three bands: top, middle, and bottom. The four glycosylation sites are shown in different colors: Blue; Site-1 (Asn 33), Red; Site-2 (Asn 108), Yellow; Site-3 (Asn 157), and Teal; Site 4 (Asn 238).

# **Supplemental Figure S6:** 3D structure of kallistatin

**Fig. S4: 3D structure of kallistatin.** The four glycosylation sites site-1 (Asn 33), site-2 (Asn 108), site-3 (Asn 157), and site 4 (Asn 238) are indicated in green. The three cysteines are indicated in yellow. The signal sequence, which is not part of the active molecule, is shown in white. The structure was retrieved from AlphaFold.com with the accession number AF-P29622-F1-v6.
